# Enhancing public sector enterprise risk management through interactive information processing

**DOI:** 10.3389/frma.2023.1239447

**Published:** 2023-12-20

**Authors:** Torben J. Andersen, Peter C. Young

**Affiliations:** ^1^Department of International Economics, Government and Business, Copenhagen Business School, Frederiksberg, Denmark; ^2^Opus College of Business, University of St. Thomas, Minneapolis, MN, United States

**Keywords:** complexity, dynamic adaptive systems, interactive information processes, public risk management, strategic risk leadership, uncertainty

## Abstract

**Introduction:**

Federal agencies are increasingly expected to adopt enterprise risk management (ERM). However, public sector adoption of ERM has typically focused on the economic efficiency of tax-financed activities based on control-based practices. This reflects an emphasis on quantifiable concerns that invariably directs attention to risk, that (by definition) relates to identifiable and measurable events, thereby downplaying uncertain and unknown aspects of public exposures. This is a potentially serious shortcoming as government entities often act as society's risk managers of last resort. When extreme events happen what were previously considered private matters can quickly turn into public obligations. Hence, there is a need for proactive assessments of the evolving public risk landscape to discern unpredictable-even unknowable-developments.

**Methods:**

The article reviews recent empirical studies on public risk management practices, effects of digitalization in public sector institutions, current strategic management research, and insights uncovered from a recent study of risk management practices in federal agencies. On this basis, the article explains how the ability to generate value from ERM can be enhanced when it intertwines with local responsive initiatives and central strategic risk analyses. It can form a dynamic adaptive risk management process where insights from dispersed actors inform updated risk analyses based on local autonomy and open exchange of information. This approach builds on specific structural features embedded in culture-driven aspirations to generate collaborative solutions. Its functional mode is an interactive control system with open discussions across levels and functions in contrast to conventional diagnostic controls that monitor predetermined key performance indicators (KPIs) and key risk indicators (KRIs).

**Findings:**

Backed by theoretical rationales and empirical research evidence, it is found that applications of ERM frameworks can produce positive results but is unable to deal with a public risk landscape characterized by uncertain unpredictable conditions with potentially extreme outcome effects. It is shown how interactive exchange of fast local insights and slow integrated strategic risk analyses supported by digitized data processing can form a dynamic adaptive system that enable public sector institutions to deal with emergent high-scale exposures. It is explained how the requirement for conducive organizational structures and supportive values require a new strategic risk leadership approach, which is contrasted to observed practices in federal agencies that are constrained by prevailing public governance requirements.

**Discussion:**

The need to deal with uncertainty and unknown conditions demands a cognitive shift in current thinking from a primary focus on risk to also appraise complexity and prepare for the unexpected where data-driven methods can uncover emergent exposures through dynamic information processing. This requires strategic risk leaders that recognize the significance of complex public exposures with many unknowns and a willingness to facilitate digitalized information processing rooted in a collaborative organizational climate. If handled properly, adoption of ERM in public risk management can consider emergent dimensions in complex public exposures applying interactive information processing as a dynamic adaptive risk management approach incorporating digitized methods to solicit collective intelligence for strategic risk updating.

## Introduction

As federal agencies implement enterprise risk management (ERM), these efforts should display the views expressed in the guidelines. This means that all material exposures to potential threats—or opportunities—should receive attention. Ample evidence suggests that this is often not the end-result. Rather, there is a tendency to focus on measurable economic effects of tax-financed public activities while passing over more uncertain and hard-to-assess exposures within a complex environment. To ameliorate this, there is a need to pay more attention to current insights from emergent developments as they occur. Here we note that the public risk landscape is distinct in terms of complexity and potential scale and scope of eventual responsibilities. Many private exposures quickly turn into public obligations when extreme events happen making governmental entities de facto risk managers of last resort. For these reasons there is a call to progress from current compliance and control-based risk management approaches toward more proactive assessments of emergent and unpredictable public exposures. To address this, the article presents a dynamic information processing approach to generate updated data-driven assessments of evolving public concerns on both measurable, and difficult-to-measure exposures.

Based on available evidence, we show that the value derived from ERM adoption can be significantly augmented if the ERM practices operate conjointly with strategic planning processes and local initiatives taken to deal with emergent conditions. This can provide a foundation for dynamic adaptive risk management where ongoing insights from operating entities update the central forward-looking analytics that inform ongoing strategic risk considerations. Dynamic adaptive risk management builds on distinct structural features including delegation of decision power, empowerment of local initiatives, and information systems for data and knowledge exchange to augment risk awareness and enable collaborative solutions.

The following section reviews current research on ERM adoption in the public sector illuminating a dearth of studies looking at uncertain and unknown aspects of the public risk landscape. Then, theoretical rationales and empirical evidence that support an interactive information processing approach are outlined as foundation for dynamic adaptive risk management where digitalized collection of local insights inform updated strategic risk assessments. Finally, we present strategic risk leadership as the fulcrum for dynamic adaptive risk management in public agencies with a view to prevailing governance and administrative constraints.

## Background

Public sector agencies have increasingly adopted enterprise risk management (ERM) often based on mandatory requirements [Office of Management and Budget (OMB), 2016; Rana et al., 2019]. Recent research shows, however, that most adoptions assume an attenuated form limited in scope and detail (Young and Hoang, 2023). Varying reasons are given for this outcome, such as difficulty obtaining leadership support, cultural barriers, organizational constraints, political considerations, lack of guidance, etc. (AFERM, 2022). Caution in implementing ERM also plays a role, frequently leading to reliance on traditional risk management methods primarily influenced by audit and internal control applications (e.g., Rana et al., 2019; Bracci et al., 2021). In many agencies, ERM is expressly seen as a management control process (Young and Hoang, 2023). There have been some notable advances and successes, but largely without paying attention to challenges related to uncertain and unknown aspects of public exposures (e.g., Bracci et al., 2021). Human behavior under conditions of uncertainty—indeed all human aspects embedded in the leader-follower dynamics—receives limited attention, which is problematic (Andersen and Young, 2020). Overcoming these stumbling blocks will require a fundamental shift in risk outlook, not only among formally appointed Chief Risk Officers (CROs), but also particularly among policymakers. It will take conscious leadership efforts to change this.

It should be noted that public sector institutions typically exist for different purposes than commercial enterprises, which presents wide-ranging and potentially extreme exposures (e.g., Fone and Young, 2000; Young et al., 2022; Andersen and Young, 2023). Yet, the transference of ERM practices developed for commercial enterprises to public sector entities rarely results in a thorough reconsideration of the underlying principles. To illustrate this commercial/public distinction, consider exposures to extreme events such as financial crises or natural, manmade, and climate-related disasters (e.g., Meyer and Kunreuther, 2017; Courbage and Golnaraghi, 2022). When these so-called low-probability high-impact incidents occur (often unexpectedly) what were previously considered private exposures frequently transform into public liabilities and governmental concerns.^1^ We know these developments all too well from recent experiences, such as, the COVID-19 pandemic, military conflict in Ukraine, climate-driven flooding events, wildfires, and similar disasters (UK Cabinet Office, 2022). In such contexts, governments and public agencies are (or become) society's risk manager of last resort with potentially unlimited exposure (Moss, 2002). These situations, however, manifest themselves outside the scope of current public risk management practices (Raine and Lloyd, 2013) and typically escape monitoring and ex post risk audits (Domokos et al., 2015).

To be clear, ERM does have a potential to advance learning from engaged discussions and create deeper insights to develop risk strategies among policymakers (Capaldo et al., 2018; Hinna et al., 2018). Yet, in practice there is limited evidence of activities beyond a control-based compliance and audit focus among public institutions (Rana et al., 2019; Bracci et al., 2021).

Therefore, this article intends to search for an approach that facilitates considerations for a wider scope of public exposures—an extensive (strategic) field—using current insights and data to update the ongoing threat assessments. This extended view, should create stronger awareness of the complex public risk environment that needs more flexible, dynamic, and adaptive approaches to navigate turbulent and rapidly changing conditions (e.g., Kay and King, 2002). It requires a different way of thinking about (public) risk management with more concern for the often unpredictable and unmeasurable effects of complex, uncertain, and unknown exposures assessing the capacity to respond to such conditions (Andersen and Young, 2020).

### Dealing with a changing environment

The ability to identify emergent risk events in advance and to quantify their impacts—as assumed in conventional risk management—does not hold in complex and uncertain settings. Under a pandemic, for example, it is not possible to make meaningful predictions to determine the societal exposures using conventional risk management tools as “*real-life complexity … escapes neat estimations by probabilistic methods*” (Seetoh et al., 2012, p. 718). As it turned out, the (global) political context was highly complex and unpredictable under COVID-19 (Shefrin, 2020). Instead, effective adaptation to extreme conditions depends on entrepreneurial improvisation where sharing of current insights creates awareness of emergent events and identifies responsive opportunities in the changing environment (Alonso et al., 2021). This approach should engage stakeholders, including frontline employees, with diverse insights rather than relying on a central risk function to assess exposures based on conventional impact-likelihood calculus (e.g., Rodriguez and Edwards, 2014).

The contemporary risk landscape is changing from probabilistic risk conditions toward uncertain and unknown circumstances with potentially extreme outcome effects (Phillips et al., 2023). These dynamic and complex environmental conditions create *wicked* problems that are multifaceted with no single optimal solution in easy sight. They comprise issues, such as, global financial crisis, food production sustainability, circular economy standards, climate change effects, and various combinatorial outcomes from these issues. The resolution to wicked problems requires open discussions and collaborative efforts where the challenge is to devise complex multi-stakeholder processes that can lever diverse contributions in collective problem analyses (Elia and Margherita, 2018). The public sector is particularly exposed to turbulent, complex, and unpredictable conditions with wicked problems that require leadership to generate viable solutions and retain the societal order in the face of disruptions.

These challenges to the public system should prompt proactive responses and adaptive processes that can uphold the essential public functions (Chen et al., 2020). This may require engagement of both internal and external competencies where information is shared in collaborative efforts to generate solutions for the whole system (Ansell et al., 2020). Indeed, an organizational capacity to process information effectively—accommodating updated insights to enlighten timely decisions—is associated with superior outcomes (Yu et al., 2019). That is, a complex crisis-situation like a pandemic calls for rapid decisions based on critical current information with frequent interactions between key decision-makers (Phillips et al., 2023). The ability to innovate and find opportunities from crisis situations can be effective when dealing with disruptive conditions (Wenzel et al., 2020) and safeguarding public assets and processes that provide essential services.

## Theoretical underpinnings

The *dynamic adaptive* risk management approach can be conceptualized through theories on strategic responsiveness, collective intelligence, and dynamic adaptation where the first two consider aspects of timely informed responses and the third explains the adaptive dynamic. In combination these theoretical lenses outline the contours of a dynamic adaptive system that—we claim—can respond effectively to ongoing environmental changes.

### Strategic responsiveness

Strategic responsiveness has been conceived as a “*bundle of capabilities to assess the environment, identify firm resources, and mobilize them in effective responsive actions with the intent of achieving strategic fit over time*” (Andersen et al., 2007, p. 410). This resonates with a “dynamic capabilities” concept based on *sensing* ongoing changes, *seizing* resources around opportunities, and *reconfiguring* organized activities as environmental conditions change (Teece et al., 1997; Teece, 2007). It is construed as complex bundles of organizational routines and processes affected by the interventions of managers and leaders (e.g., Teece et al., 2016; Teece, 2018). It can be displayed as strategy-making where managers decide on responsive initiatives as they observe ongoing changes and adjust activities to improve performance (Andersen and Bettis, 2015). Different response capabilities across firms that operate in similar contexts affect their relative effectiveness where high performing organizations generate more stable outcomes above average performance (Andersen et al., 2007). This effect can be modeled as an adaptation process where the ability to observe developments and learning to generate better opportunistic responses lead to more adaptive strategic decisions.

The updated versions of major ERM frameworks emphasize similar associations to the strategy-making process—or strategy-setting (e.g., COSO, 2017; ISO., 2018)^2^ stating that risk assessment is an important precursor for making strategic decisions. However, this intended aim is rarely achieved even though proclaimed to offer the highest potential for ERM improvement (Viscelli et al., 2016). So, risk management and strategic planning continue to be performed as compartmentalized activities in many private and public organizations. Yet, the empirical evidence shows that planning, i.e., a rational analytical approach to strategy-making, can have a significant positive and material mediating effect on ERM performance (e.g., Sax and Andersen, 2019) where innovative responses are induced by strategic planning (Sakellarios et al., 2022).

### Collective intelligence

Collective intelligence is conceived as distributed group intelligence where knowledge resides with many individuals across a social system that holds relevant experiential insights from the interactions performed among various stakeholders. Pentland (2007) demonstrated how social networks provide a basis to understand human intelligence beyond the common focus on individuals in cognitive science as they form a collective “network intelligence” mediated by communication processes. It is often seen as an IT-enhanced phenomenon where the intelligence subsumed in individual skills is mobilized in real-time digitized form through computer intervention (Lévy, 1999). Lévy (2010) further argues that digital technologies provide powerful tools to augment the joint cognitive processes and multiply the formation of collective intelligence through information exchange within digitalized networks.

Communication and information technology (CIT) can connect wide networks of individuals and facilitate interaction across time and space forming a joint virtual knowledge base. The current insights of many individuals constitute their “collective wisdom” that can generate more accurate forecasts (Surowiecki, 2004; Page, 2007a) if the group includes diverse independent individuals with relevant expertise (Hong and Page, 2004; Page, 2007b). It can also increase the joint ability to perform specific tasks (Woolley et al., 2010, 2015) amplified by social sensitivity and broad participation. Use of collective intelligence is generally effective when adapting to uncertain environments facilitated in different domains from computer enhanced networks to agent-based systems (Schut, 2010). An elaborated definition refers to “*the capacity of human collectives to engage in intellectual cooperation in order to create, innovate and invent*” (Lévy, 2010, p. 71), i.e., it can improve resolutions to complex issues by enhancing an innovative capacity. This is an important design element in effective learning organizations that can generate collaborative solutions to wicked problems (Kirschner et al., 2009; Zambrano et al., 2019). These network structures resemble the collective ideation processes associated with “swarm intelligence” where creative solutions are advanced through open sharing of information and ideas (Gloor, 2006, 2011, 2017; Malone, 2018).

### Dynamic adaptation

Dynamic adaptation derives from ideas of combined fast and slow information processing that stimulates an ability to deal with turbulent unpredictable contexts advancing activities in non-linear ways through interim meta-stable positions (e.g., Kelso and Engstrøm, 2006; Pfeifer and Bongard, 2006). This resembles the dynamic models of the human brain developed in modern cognitive science (e.g., Kahneman, 2011). The human brain, groups of people, organizations, and societies can be interpreted as fast-slow information processing systems, where fast insights from current responsive actions inform slow comprehensive forward-looking analyses (e.g., Andersen and Fredens, 2013; Andersen and Hallin, 2016). In organizations, the ongoing learning from emerging strategic initiatives in the operating entities and the strategic planning considerations at head office constitute fast and slow information processes (e.g., Andersen, 2013, 2015). The fast-slow interactive information processing has adaptive advantages but also some limitations caused by potentially distorting biases (Schwenk, 1984; Finkelstein, 2003; e.g., Bazerman and Watkins, 2008; Heffernan, 2011; Shefrin, 2016) and cognitive filters (e.g., Reitzig and Sorenson, 2013; Tuckett and Nikolic, 2017; Sokol-Hessner and Rutledge, 2018). To the extent these adverse influences can be subdued, using (fast) experiential insights from local responses to inform and update the (slow) analytical strategic planning rationales can form a dynamic adaptive system (Andersen, 2015; Andersen et al., 2018). It can exploit the entrepreneurial minds of many internal stakeholders dispersed throughout the organization where their diverse insights can support the adaptive thinking of central strategic decision-makers.

The ability to deal with disruptive events and create adaptive outcomes depends on a capacity to generate innovative responses and using them to modify organizational, or social activities to emergent changes through integrative reconfiguration mechanisms (Teece et al., 1997; Andersen et al., 2007). From this perspective, the empirical evidence on the value creating potential of ERM in turbulent contexts remains rather elusive (Andersen and Sax, 2020). Conversely, a recent study shows that ERM performance can be (significantly and materially) enhanced by decentralized response initiatives in conjunction with central strategic planning processes (Andersen et al., 2022). The study measures ERM adoption by the extent to which the organizations follow the procedures prescribed by the major frameworks (COSO, 2004; ISO., 2009), i.e., whether they abide by the basic principles of ERM (Andersen et al., 2022). So, adhering to the principles of ERM can create value but the performance effects are significantly enhanced when combined with more complex bundles of dynamic adaptive capabilities (Peteraf et al., 2013). Instituting this in organizations requires leadership that is attentive to the dynamic adaptive design parameters of power delegation, open information exchange, and collaboration where managers can overrule formal routines as needed (Teece et al., 2016).

### Implications

The complementary perspectives that outline a model of adaptive strategy-making and dynamic risk analysis have not been formally specified despite extensive effort (Schilke et al., 2018). This model resembles collaborative approaches applied in investment planning where internal groups engage in the strategizing efforts soliciting current insights and tacit knowledge from diverse participants (Weigand et al., 2014). It also embraces ideas of open strategy-making where interested stakeholders can contribute through online platforms using diverse insights from *ad hoc* crowds (Malhotra et al., 2017).

The interactive information processing logic is deceptively simple and consistent with recognized perspectives on interactive strategy-making (e.g., Mintzberg and Waters, 1985; Burgelman and Grove, 2007). However, the model contravenes a common belief that the ability to manage major risks requires central control-based approaches and that comply with a predetermined risk appetite statement condoned by top-management. Information processing in social systems also has behavioral and ethical dimensions that can affect outcomes and therefore must be considered (e.g., Andersen and Young, 2020). The fast-slow interaction implies communication between individuals located in different parts of an organization, or social system, including executives, operating managers, and employees. CIT can facilitate exchange of data across time and space and possibly be devised in ways that may reduce the adverse effects of potential information filtering (Gleasure and Feller, 2016, 2018; e.g., Brooks, 2017).

## Model development

**Figure 1:**
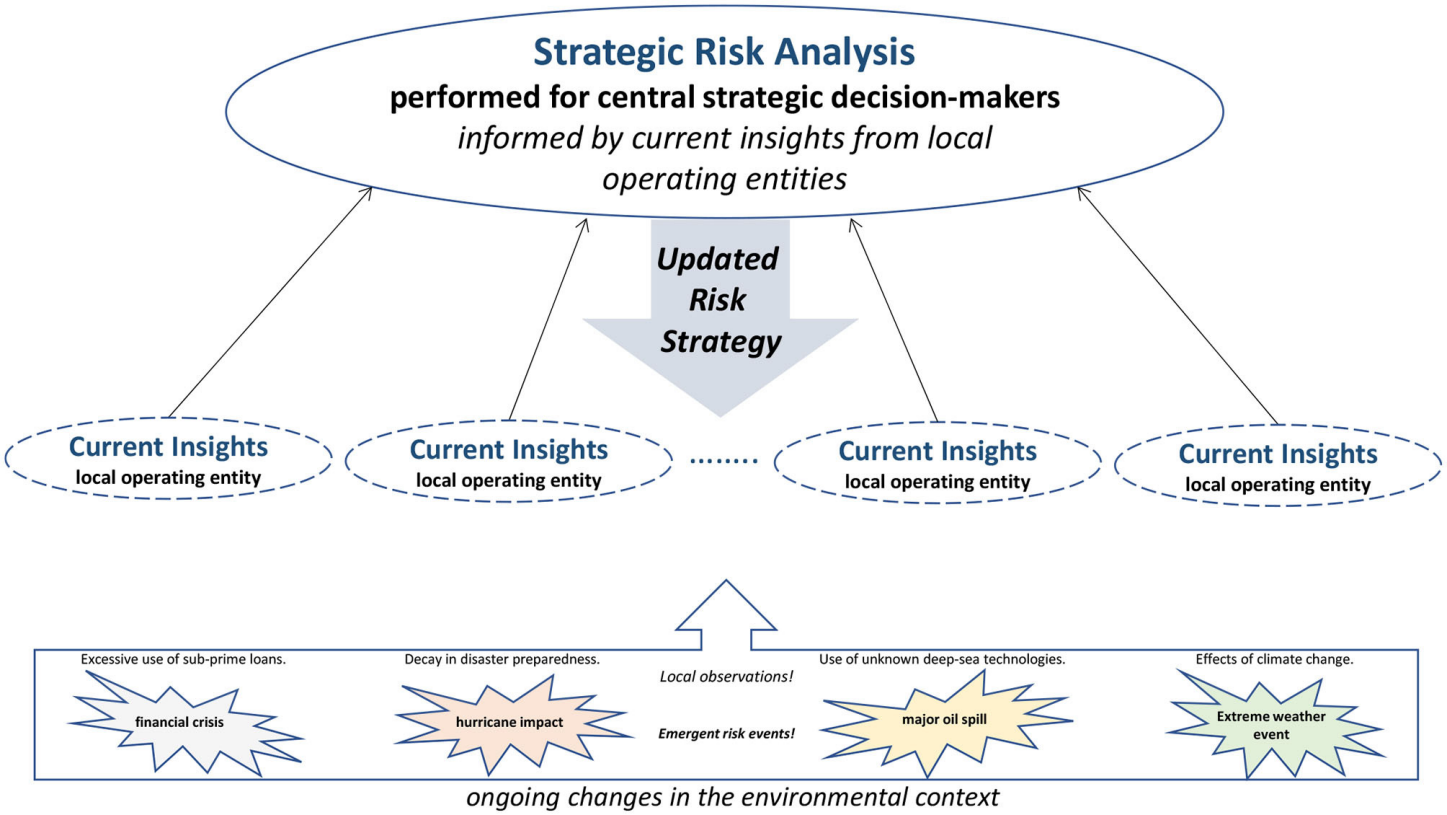
Interactive information processing to update the risk strategy.

Dynamic response capabilities embedded across various operating entities provide strategic decision-makers in an organization, or public policymakers within a social structure,^3^ with first-hand insights to sense impending changes and identify opportunities that may deal with emergent events (Teece, 2007; e.g., Andersen et al., 2007). Getting access to current insights about evolving developments, and how the operating entities respond to them can update adaptive risk strategies and point to viable solutions. The ability to use these insights depends on a willingness among central strategic thinkers, decision-makers, or leaders, to consciously collect and consider the knowledge held by individuals in the frontline and use this intelligence to inform the strategy deliberations (Figure 1). A function for Strategic Risk Analysis performs ongoing risk assessments for the central strategic decision-makers informed by Current Insights from local operating entities to generate an Updated Risk Strategy.

However, strategic decision-makers often ignore or remain unaware of emergent risk events revealed by insights from frontline employees in the operating entities often due to the influence of “dominating elitist” connections (Chen et al., 2009). To improve the ability to recognize new developments, the central risk leaders should probably increase their interactions with frontline personnel building social relationships with lower-level managers and employees. The important updated information can be generated through systematic collection and analysis of current insights from frontline actors in the operating entities (e.g., Andersen, 2015; Andersen and Hallin, 2017). They may also be extracted from direct face-to-face discussions between executives and operating managers in interactive control processes (e.g., Simons, 2005; Andersen and Sax, 2023). The insights gained from responses to evolving changes in the local task environments can be used in a collaborative learning process where an analytical strategic risk function interacts with the operating entities in open exchange of information.

### Supporting collaborative effort

People in the operating entities gain updated insights as they observe and respond to often subtle changes in their immediate surroundings as they interact with colleagues, customers or clients, and partners to complete their daily routines and activities. The observed changes in the environment may have strategic significance where systematic data collection and analysis of insights can provide important information for strategic decision-makers as they consider the needs for adaptive actions in an updated risk strategy. But strategic decision-makers develop “dominant logics” formed by past experiences and create cognitive biases that influence the interpretation of observed developments and emergent risk scenarios (e.g., Bettis and Prahalad, 1995; Bazerman and Moore, 2009). Many strategic decision-makers also subconsciously hesitate to consider information from lower-level operating entities due to cognitive and power-related biases (e.g., Dutton, 1993; Blader and Chen, 2012; Tost et al., 2013).

The ability to update strategic decision-makers and public policymakers with current insights from the operating entities is essential for the ability to develop effective adaptive organizations and societies. It is important that ongoing strategic thinking is informed by current insights generated as the local operating entities act on emerging changes and learn from events as they evolve. However, the interactive information exchange of current insights from operating entities and updated risk strategies from a central risk analytical function relies on a willingness among strategic decision-makers to use information from lower-level employees. The positive benefits from collaborative learning can only unfold when the current insights from local responses inform the assessments for updated adaptive risk strategies.

The collection of such “weak signals” may be enhanced by environmental scanning processes, solicitation of expert panels, and data collection using different internet-based tools from networks of creative individuals with intuitive skills and broad knowledge (Holopainen and Toivonen, 2012). IT and digital capabilities can be applied to exchange data that capture ongoing changes and present possible ways to respond (Overby et al., 2006). CIT can give access to relevant market intelligence including data from adjacent governmental units that may reveal important public preferences, political initiatives, etc. Similarly, a range of digital solutions are available to facilitate idea generation, crowdsource solutions, structure communication platforms, and more to support collaborative efforts.

### Dynamic information processing

The ability to generate innovative solutions is superior in multi-actor collaborative networks compared to hierarchical and market-based approaches. The positive innovation results can be derived from collaboration in networks that integrate different partnerships including swarms of public and private actors (e.g., Gloor, 2006, 2011, 2017). While collaborative innovation building on diverse contributions to generate public value is superior, the ability to manage diversity requires a common purpose whereby participating actors can process their contributions (Torfing and Ansell, 2017; Torfing et al., 2020). This ascribes to a need for slow analytical information processing linked to strategic risk assessments based on generally accepted objectives and goals. It is argued that eristic reasoning based on debate and exchange of arguments is required to deal with extreme uncertainty whereas heuristic reasoning, i.e., learning through trial-and-error responses is insufficient on its own (Kurdoglu et al., 2022). The interactive information processing model engages both heuristic and eristic reasoning to deal effectively with unpredictable complex conditions. In uncertain evolving environments with many unknowns, the only way to “find out” is by trying, or responding, but this also requires a sensemaking process where the updated trial-and-error learnings can be interpreted to form a new understanding of the evolving context. This is the underlying dynamic of the interactive information processing approach. However, it requires leadership to set up structures that enable, support, promote, and encourage collaboration that can foster collective learning for creative problem solving.

### A need for strategic risk leadership

The interactive information processing model implies open communication and data exchange across levels and functions where dominant connections with executive peers lead to different perceptions of the environment than contacts to individuals with a lower hierarchical status. Hence, maintaining nearly exclusive associations with other elites limits the peripheral vision of executive decision-makers (Chen et al., 2009). In contrast, Nonaka et al. (2016) present leadership approaches more likely to foster dynamic response capabilities based on team-level interactions among individual members in the organization. They argue that sensing of environmental changes predominantly is done by frontline employees where the utility of their collective insights must be supported by leadership to unleash the potential to generate adaptive innovation (Nonaka et al., 2016). This involves encouraging and providing a space for new ideas among entrepreneurial individuals in response to tensions between existing operating systems and changing requirements in a rapidly changing environment. The leaders must enable a capacity to deal with potential operating and servicing shortfalls and foster creative solutions for adaptive initiatives (Uhl-Bien and Arena, 2018). Conversely, mimetic strategic behaviors and normative pressures can lead executives toward misguided decisions. Hence, organizations with tightly connected boards fare worse during economic recession compared to firms with less connected boards that are less exposed to pressures of common beliefs (Hudson and Morgan, 2022). It takes a high degree of consciousness among individual leaders to recognize these potential biases and take the necessary measures to circumvent them.

In rapidly changing contexts, leaders are challenged to adapt organizational activities to remain relevant and effective. An uncertain environment with many unknowns requires an ability to deal with potential threats and explore adaptive opportunities. While uncertainty often triggers adoption of control-based approaches with more reporting and constraints, this is a self-defeating proposition as tight controls reduce flexibility and experimentation for creative solutions. Prioritizing immediate efficiencies tend to limit the possibility to create responsive opportunities with a long-term value potential. It is not possible to measure and control things in uncertain unpredictable contexts whereas experimentation and learning from responsive initiatives to deal with evolving developments can generate workable solutions.

It takes a certain leadership style to recognize the rich, diverse, and deep insights that reside among individuals within and around the organization. A *transformative* leadership style breaks with the control-based approach to engage people in generating opportunities to achieve higher-order goals (Bass and Riggio, 2006; Borener et al., 2007). A *respectful* leadership style can encourage knowledge sharing through social mindfulness with a willingness to increase opportunities for others in open exchange of information (Gerpott et al., 2020). An *enactive* leadership style acknowledges the limitations of (even powerful) executives and seeks insights from many individuals in the organization as new knowledge is formed through concrete interactions with the changing environment (e.g., Varela et al., 1992; Thompson, 2007). Meaning is generated from ongoing interpretations of responses and reactions experienced through actions taken as the environmental conditions evolve and derives from “*the interplay of action and interpretation rather than the influence of evaluation on choice*” (Weick et al., 2005, p. 409). So, *enactive* leaders form their understanding of the environment from guided responses to ongoing environmental changes where cognitive structures are updated by observed reactions and outcomes.

In unpredictable contexts effective risk management requires *collaborative* leadership (Jacklin-Jarvis and Potter, 2020) to facilitate collective search for solutions across organizational networks (Kim et al., 2021). It motivates and encourages engagement rather than it directs and controls activities, and it pays attention to behavioral aspects that facilitate information sharing and generate innovative ways to adapt (Murphy et al., 2017; Kapucu and Ustun, 2018). There is a need for *strategic risk leadership* with a moral connection between leaders, employees, and related stakeholders recognizing that uncertain and unknown environmental factors are important (Andersen and Young, 2020). Strategic risk leaders think critically about the (potential) exposures and effects of dynamic complex contexts where outcomes are hard to predict and require collaborative efforts to generate sustainable solutions.

### Organizational culture

The presence of supportive cultural values is essential to execute organizational processes including those promoted by ERM that often refer to “risk-aware” cultures—presumably hinting that organizational members should be conscious about possible exposures. Yet, the implementation of standardized routines may turn into bureaucratic practices with limited awareness of emergent risk events and a reduced capacity to respond proactively to ongoing changes. Responsiveness calls for norms of open communication around early warning signals with joint efforts to gain deeper understanding and generate effective solutions. Establishing these supportive values derive from leadership priorities led by example—not by words—aligned with supportive decision-structures, information systems, and incentive schemes. As Miller (2022) argues: “*the responses and behavior of senior leaders, middle management, and frontline personnel … are far more indicative of the risk culture than any number of policies and memorandums*”.

The empirical evidence suggests that organic structures where people have room to improvise, compared to more mechanistic structures, make faster and better progress toward ERM adoption (Kimbrough and Componation, 2009). However, leaders (CFOs for instance) that front the ERM efforts are more attuned to the formal strategy setting views of ERM than the employees that operate the ERM frameworks (Viscelli et al., 2016). So, there are significant discrepancies between executives and employees where senior leaders typically have a much rosier perception of the risk culture than the staff that executes the ERM practices (Sheedy and Griffin, 2018). Nonetheless, a recent study shows that a compliance-based defensive organization can adopt a more cognitive and engaged risk climate (Agarwal and Kallapur, 2018). Conscious monitoring of behavioral metrics can proactively guide the risk management policies where associated training programs may develop a risk-aware environment with conscious strategic decision-makers (Miller, 2022). It is relevant to know if the risk climate in the organization lives up to the ERM expectations regarding “*ethical values, desired behaviors, and understanding of risk in the entity*” (COSO, 2017). This concern should be extended to consider the structural dimensions of dynamic adaptive risk management with interactive information processing capabilities across many individuals in and around the implicated organizational entities.

## Discussion

The preceding review of current research shows that ERM adoption in the public sector remains focused on well-intended motives to increase the efficiency of public operations but downplays concerns about major exposures to uncertainty and the unknown. ERM attempts to deal proactively with foreseeable risk scenarios—but it is less successful in addressing the (often) unforeseen and (potentially) extreme public risk exposures. The ERM practices—as generally employed—are insufficient to deal with a complex public risk landscape with major events like financial crises, pandemics, military conflicts, climate-related events, etc. More is required to deal effectively with this evolving public risk environment.

A recent study of ERM adoption in US federal agencies (Young and Hoang, 2023) examines *leadership readiness* to better understand how risk leaders translate the specifications by the Office of Management and Budget (OMB) (2016) A-123 Circular to their organizations. This provides specific insights into the perceived readiness of risk leaders including their considerations about leadership issues, agency work, organizational culture, employee awareness, and understanding of ERM principles. Three internal factors emerge from this inquiry as critical obstacles to successful ERM adoption that corroborate the extant literature.

### Leadership short-termism

The tenure of political leadership is subject to political cycles and career considerations that tend toward shorter terms of service for administrative leaders in federal agencies where major initiatives, like ERM adoption require longer-term leadership commitment. So, short-termism in the leadership ranks is an obstacle that puts a premium on risk leaders (however designated) with knowledge, skills, and acuity to act “as if” a sustained top-level commitment is present and maintained.

### Organizational cultural barriers

Difficulties in aligning ERM with organizational culture (or vice versa) are often cited as challenges to implementation where short-termism in leadership makes it harder to induce cultural changes. Even with a formally designated (long-term) risk leader, the relative standing of that individual might restrain the cultural change efforts.

### Legal and political constructions

The legal and political considerations in federal agencies may be at odds with conventional ERM practices as well as many behavioral factors that influence performance outcomes. The principle of power separation may conflict with efficiency/efficacy objectives, privacy concerns can delimit information-sharing, budgeting processes can interfere with longer-term considerations, while the political dynamic often introduces major policy changes, etc. For these reasons public sector risk management is materially distinct from commercial ERM practices (Andersen and Young, 2023), which suggests that ERM practices cannot be transferred wholesale into public agency settings.

The distinct public sector “conditions” should be considered when proposing a dynamic adaptive risk management approach based on principles of interactive information processing based on data exchanges among diverse stakeholders across levels, functions, and entities. In a public context, the core idea is to quickly uncover current insights generated at the operating frontlines where local agents are the first to observe new developments. Acting as first-responders help agents learn as they gain current insights from initial responses to the evolving changes. Collecting this information and feeding it into analytical sensemaking processes around strategists and policymakers can inform updated risk strategy reviews considering opportunistic solutions to needed adaptations based on collective efforts among engaged stakeholders.

This approach requires a leadership style with a willingness to engage the collective intelligence to develop better updated risk strategies. It can use various CIT systems and AI tools to facilitate the data and information exchange processes while circumventing effects of information filtering and cognitive biases. This should not be done uncritically as use of computer-based information networks raises concerns that require further research to untangle including issues of privacy, transparency, manipulation, etc. While there are benefits to derive from AI enabled information processing, it should be adopted in ways that aligning with required process specifications and network arrangements (e.g., Taylor, 2019). It must also recognize how AI technology interacts with people and affects social institutions to ensure that norms formalized in the digitalized systems align with the values of the social settings where they are deployed (Venkatasubramanian et al., 2020). As important governance functions can become informed—even automatically executed—by machine learning algorithms (for the claimed benefit of society), there is a need to impose human oversight functions that can secure privacy and fundamental rights of citizens (Florin, 2022). Hence, the nationwide network of fusion centers supported by the Department of Homeland Security (DHS) to collect risk-related data failed to ensure proper use and thereby have undermined civil rights and liberties under the guise of public security (German et al., 2022).

It is argued that innovation emerges from disorder through “destructive creation” that may lead to “creative destruction” as successful innovations are diffused throughout the organization (Li et al., 2020). Yet, the most influential innovative ideas arise from in-person contacts across diverse entities suggesting that collaboration through technological connectivity may enable short-term innovations that eventually collapse due to cultural and psychological factors (Li et al., 2020). The use of videoconferencing is also found to inhibit creative ideas as this form of communication misses essential features of in-person interactions, such as, eye-gaze, recall stimuli, and latent semantics (Brucks and Levav, 2022). Hence, applications of CIT and AI to build interactive relationships, facilitate information exchange, and generate innovative solutions are promising but most likely cannot stand on their own.

Empirical studies of digitization,^4^ or digitalization, typically focus on positive aspects and discards negative implications (Trittin-Ulbrich et al., 2021), such as, professional concerns of misapplied digital outputs (Karsten, 2021) or subversive actions to regain work discretion (Lammi, 2021). Digitalization affects relationships and administrative workflows where adoption of digital technology is shaped by discourse influenced by vested interests, ethical concerns, and algorithms with outcomes that may threaten professional autonomy and reduce service quality (Andersson et al., 2022). In this context, studies on implementation of digital solutions are often vague and incomplete (Carroll et al., 2023). Hence, implementation of digital technologies can depersonalize work with negative effects on the organizational climate while interactive employee–manager relationships and involvement reduce these adverse outcomes (Palumbo, 2022). Failed digital transformations are often caused by “blind” pursuit of an “exciting” technology without a clear purpose, which instead calls for a *human* leadership-centric approach to foster innovative adaptation based on insights from many individuals (Kotter et al., 2021). This requires an open engaging leadership style where change derives from interaction among many people in the organization and external stakeholders to generate opportunities for adaptive moves (Kotter et al., 2021). That is, leadership makes the difference.

This makes it clear that development of dynamic adaptive systems not only calls for different digital technologies, including CIT and AI enhanced tools, but ultimately requires a different type of leader and a specific leadership style—*strategic risk leadership*. This requires further—and deeper—investigation where it is fair to acknowledge that, among the many challenges is also one of creating organizational “buy-in” and cultural harmony. The aim of achieving these goals—if even possible—tends to dominate the first years of a CRO's tenure (University of St. Thomas, 2014). Thus, alongside the task of imposing dynamic, or adaptive response capabilities—we see significant related leadership challenges. It seems critical that leaders adopt more interactive and collaborative approaches guided by “strategic” sensibility for long-term resilient outcomes. This can be learned, but success is greatly influenced by individual personality traits, attitudes, and experiences. Other complications, as noted, include short-termism in federal agencies, along with the political dynamics and public budget cycles. The leadership of cultural change raises other concerns. The open sharing of information requires formal permissions and can lead to potential criticisms—where many risk issues imply potential errors and mistakes—particularly under extreme uncertainty—that may cause legal actions, ethical concerns, and political criticisms.

So, political acuity and administratve acumen would seem important attributes among risk leaders in public agencies—on top of leadership styles that differ from conventional control-based management approaches. It appears likely that formally appointed risk leaders must possess rather distinct capabilities—at times requiring them to lead absent engagement from executive directors, or political appointees, with hard-to-change cultural obstacles, and few possibilities to circumvent the unique pressures of public policy.

## Conclusion

A thorough review of empirical studies points to dynamic adaptive risk management based on interactive information processing as a promising way of upgrading ERM to deal with uncertainty and unknown exposures in the evolving public risk landscape. CIT and AI can facilitate the open information exchange in ways that avoid cognitive biases, filtering effects, and data manipulation but more research is needed to uncover these possibilities. The most updated empirics on digitization in the public sector shows that positive economic effects can be counteracted by unexpected organizational and social consequences. Hence, it takes a particular leadership style to foster organizational structures that enable entrepreneurial initiatives supported by values conducive to collaborative efforts with open information exchange and communication. It requires leadership skills rather than technical prowess with a different way of thinking about public risk management issues. It is reflected in *strategic risk leadership*—a mental shift recognizing the importance of uncertainty and unknowability while thinking critically about emergent events in dynamic complex public contexts showing a willingness to engage collective intelligence and collaborative efforts in the development of responsive risk strategies.

## Data availability statement

The original contributions presented in the study are included in the article/supplementary material, further inquiries can be directed to the corresponding author.

## Author contributions

All authors listed have made a substantial, direct, and intellectual contribution to the work and approved it for publication.

## Footnotes

^1^To illustrate this, President Bush as an example signed a US$ 10.5 billion relief package to support relief efforts around New Orleans after Hurricane Katrina's landfall in 2005. Treasury Secretary, Paulson and President Bush also proposed buying up to US$ 700 billion mortgage-backed assets from financial institutions in fall 2008 to improve market conditions. In early 2009, the Obama administration proposed an economic stimulus package at an estimated cost around US$ 825 billion and after the Deepwater Horizon explosion in 2010, the federal authorities committed resources (working around the clock) to mitigate the impact of the oil spill. In short, central government and its agencies typically pitch in with substantial resources, when extreme, but in principle foreseeable events happen.

^2^The ERM frameworks use the term, “*strategy setting*”, to denote the efforts to formulate, or develop a formal strategy for intended activities.

^3^Defined as the distinctive arrangement of institutions set up for citizens to interact and operate in a society.

^4^Use of digital technologies implies “*digitization*” that converts information from paper-based to digital storage, and “*digitalization*” that (supposedly) deals with the conversion of manual workflows to digital processes. However, we find that work practices in the public sector change with digitization (Plesner et al., 2018), so the distinction between the two terms is truly blurred.
